# Deployment experiences of military nurses: A systematic review and qualitative meta‐synthesis

**DOI:** 10.1111/jonm.13201

**Published:** 2020-11-20

**Authors:** Huijuan Ma, Jinyu Huang, Yajie Deng, Yue Zhang, Fang Lu, Yuhui Yang, Yu Luo

**Affiliations:** ^1^ School of Nursing Third Military Medical University Army Medical University Chongqing China; ^2^ McGill University Montreal QC Canada; ^3^ Department of Military Nursing NCO School Army Medical University Shijiazhuang China

## Abstract

**Aims:**

The purpose of this systematic review is to explore military nurses’ preparation, deployment and reintegration experiences in order to provide recommendations for effective management of the nursing team.

**Background:**

Nurses provide health care in different settings including community, hospital and the disaster site. Military nurses have a long history of deploying for global health.

**Method:**

A systematic review and qualitative meta‐synthesis of studies focusing on the preparation, deployment and reintegration experiences of military nurses was carried out.

**Results:**

Five synthesized findings were concluded: (a) preparing and sharing experience are the key coping strategies; (b) transition from the civilian care to emergency situations; (c) teamwork contributing to team bonding and the growing role of nursing in the medical team; (d) devoting to nursing duty achieves growth; (e) reintegration is not easy and external support matters.

**Conclusion:**

Transition from civilian care to deployment and from structured deployment environment to reintegration poses challenges to nurses, and better preparation, sufficient support enables them to gain growth.

**Implications for Nursing Management:**

Nurse managers should consider how to sustain a competent and ready nursing team by proposing training protocols to nurses for the potential challenges during the deployment cycle when responding to disasters and public emergencies.

## 1 INTRODUCTION

The four fundamental responsibilities of nurses are to promote health, to prevent illness, to restore health and to alleviate suffering (International Council of Nurses, 2012). Nurses belong to the principle group delivering health care service, not only in primary care and hospital, but also in war and non‐war humanitarian aid work. Nurses engage in disaster relief, public emergencies response and United Nations peacekeeping operations where they save lives and exhibit the professionalism of nursing.

The ever‐changing health care environment and the increasing health care demands have put more strains on nurses in the front line. It is reported that nurses are more likely to burn out and leave due to intense pressure, heavy workload, frequent work shift and inadequate relax time (Adriaenssens et al., 2015). Compared to the routine care in primary care or hospital, nursing contexts during deployment such as war, humanitarian aid, and public emergencies pose more challenges for nurses. These challenges are normally characterized by dangerous environment, heavy workload, and severe patients, and thus, nurses have higher intention to leave after the deployment (Cox et al., 2010). Therefore, scientific management is necessary to maintain a stable and competent nursing team. Meanwhile, the management shall cover the whole period of deployment, which is composed of the preparation before departure, the deployment and the reintegration.

There has been a long history of military nurses engaging in war, military operations and humanitarian missions. Military nurses normally work in military clinics and hospitals after having a bachelor's degree in nursing, an RN licence and military training as well as joining military nursing commission. Governments in many countries prefer to deploy military capabilities for global health (Michaud et al., 2019). Military nurses all over the world have been deployed alongside their armies to provide health care during conflicts or to provide humanitarian aid in response to disasters, public emergencies and epidemics (Chambers, 2011; Lu et al., 2016).

There are many studies reporting either the preparation, the deployment or the reintegration experience of military nurses during war or non‐war military missions. However, no systematic review has been done from the perspective of these three phases of deployment together. Therefore, this paper summarizes and synthesizes reliable qualitative evidences exploring the preparation, the deployment and the reintegration experiences of military nurses in order to fully understand the challenges they are facing and thereby provide recommendations for effective management of the nursing team.

### 1.1 Aim

The objective of this review was to identify and synthesize the available qualitative evidence about the preparation, the deployment and the reintegration of military nurses.

## 2 METHODS

### 2.1 Research design

Meta‐aggregation approach developed by Joanna Briggs Institution (JBI) was used in this systematic review and qualitative meta‐synthesis. This approach is grounded in pragmatism and phenomenology to assist synthesis of qualitative studies (Lockwood et al., 2015). This research work was carried out between October 2019 and September 2020.

### 2.2 Search strategy

A three‐step strategy was utilized in this review. First, an initial limited search of CINAHL was undertaken followed by a text word analysis of the title, the abstract and the index term used to describe an article. Second, a search using all identified keywords and index terms was undertaken across all included databases (PubMed, CINAHL, EMBASE, PsycINFO, Cochrane Library). Third, the reference lists of all identified reports and articles were searched. Only those studies published in English were considered in this review, and there was no restriction in the publish date because studying the deployment experience of different time would provide a comprehensive understanding of this topic. The full search strategy is provided in Appendix S1.

### 2.3 Inclusion criteria

#### 2.3.1 Types of participants

This review considered studies focusing on licensed military nurses who are contracted to provide medical care specifically to patients in hospitals from the major branch of the military, including the Army, the Navy and the Air Force.

#### 2.3.2 Phenomena of interest

The phenomena of interest for this review were experiences of military nurses who were deployed to a different place to provide health care services. These included, but were not limited to, the experiences of deployment to war or non‐war military operations, disasters, public emergencies and epidemics. The phenomena of interest also covered experiences of the preparation before departure and the reintegration after deployment.

#### 2.3.3 Context

This review considered studies that explored the experience of military nurses during deployment cycle. These deployment settings were in any country, cultural context or geographical location.

#### 2.3.4 Types of studies

This review considered studies that focused on qualitative evidences including designs based on phenomenology, grounded theory, ethnography, etc.

### 2.4 Assessment of methodological quality

Qualitative papers selected for retrieval were assessed by two independent reviewers for methodological validity prior to inclusion in the review using JBI Qualitative Critical Appraisal Checklist. This checklist outlined 10 criteria that aim to establish the appropriateness of the methodological approach, the method application and the representation of the voices of participants in studies (Table 1). All disagreements between the reviewers were resolved after discussing with a third reviewer.

**Table 1:** Assessment of methodological quality

Citation	Q1	Q2	Q3	Q4	Q5	Q6	Q7	Q8	Q9	Q10
Han (2019)	Y	Y	Y	Y	Y	N	Y	Y	Y	Y
Conlon et al. (2019)	Y	Y	Y	Y	Y	N	Y	Y	Y	Y
Rivers et al. (2017)	Y	Y	Y	Y	Y	N	N	Y	N	Y
Peyrovi et al. (2015)	Y	Y	Y	Y	Y	N	N	Y	Y	Y
Elliott (2015)	Y	Y	Y	Y	Y	N	Y	Y	Y	Y
Ekfeldt et al. (2015)	Y	Y	Y	Y	Y	N	N	Y	Y	Y
Doherty and Scannell‐Desch (2015)	Y	Y	Y	Y	Y	N	Y	Y	N	Y
Rivers et al., (2013)	Y	Y	Y	Y	Y	N	N	Y	Y	Y
Goodman et al., (2013)	Y	Y	Y	Y	Y	N	Y	Y	N	Y
Scannell‐Desch and Doherty (2010)	Y	Y	Y	Y	Y	N	Y	Y	N	Y
Agazio (2010)	Y	Y	Y	Y	Y	N	N	Y	Y	Y
Rushton et al. (2008)	Y	Y	Y	Y	Y	N	Y	Y	Y	Y
Griffiths and Jasper (2008)	Y	Y	Y	Y	Y	N	N	Y	Y	Y
Scannell‐Desch (2005)	Y	Y	Y	Y	Y	N	Y	Y	Y	Y
Cox (2005)	Y	Y	Y	Y	Y	N	Y	Y	N	Y
Scannell‐Desch (1996)	Y	Y	Y	Y	Y	N	N	Y	N	Y

*Source:* Aromataris and Munn (2020)

Q1: Is there congruity between the stated philosophical perspective and the research methodology? Q2: Is there congruity between the research methodology and the research question or objectives? Q3: Is there congruity between the research methodology and the methods used to collect data? Q4: Is there congruity between the research methodology and the representation and analysis of data? Q5: Is there congruity between the research methodology and the interpretation of results? Q6: Is there a statement locating the researcher culturally or theoretically? Q7: Is the influence of the researcher on the research, and vice versa, addressed? Q8: Are participants, and their voices, adequately represented? Q9: Is the research ethical according to current criteria or, for recent studies, and is there evidence of ethical approval by an appropriate body? Q10: Do the conclusions drawn in the research report flow from the analysis, or interpretation, of the data?

### 2.5 Data extraction and synthesis

Qualitative data were extracted from papers included in the review using the JBI Qualitative Assessment and Review Instrument (QARI) Data Extraction Tool for Qualitative Research. Data extraction is a multi‐phase process including extracting general details of papers, extracting findings and allocating level of “Credibility” for each finding. General details of papers were composed by author, published year, methodology, methods, phenomena of interest, the setting of the research, demographics of participants, data analysis and conclusion (Appendix S2). Levels of “Credibility” were allocated on reviewers’ perception of the level of support with the illustration selected from the study. These findings were rated with the three levels of credibility (Unequivocal, Credible or Unsupported). “Unequivocal” relates to findings that are beyond reasonable doubt, and “Credible” means that the findings are deemed plausible. Findings rated as “Unsupported” were excluded.

Data synthesis was undertaken with three‐step process that was extracting all findings from all included papers with illustrations and levels of “Credibility”, developing categories for findings and producing a comprehensive set of synthesized findings.

## 3 FINDINGS

### 3.1 Study inclusion

**Figure 1:**
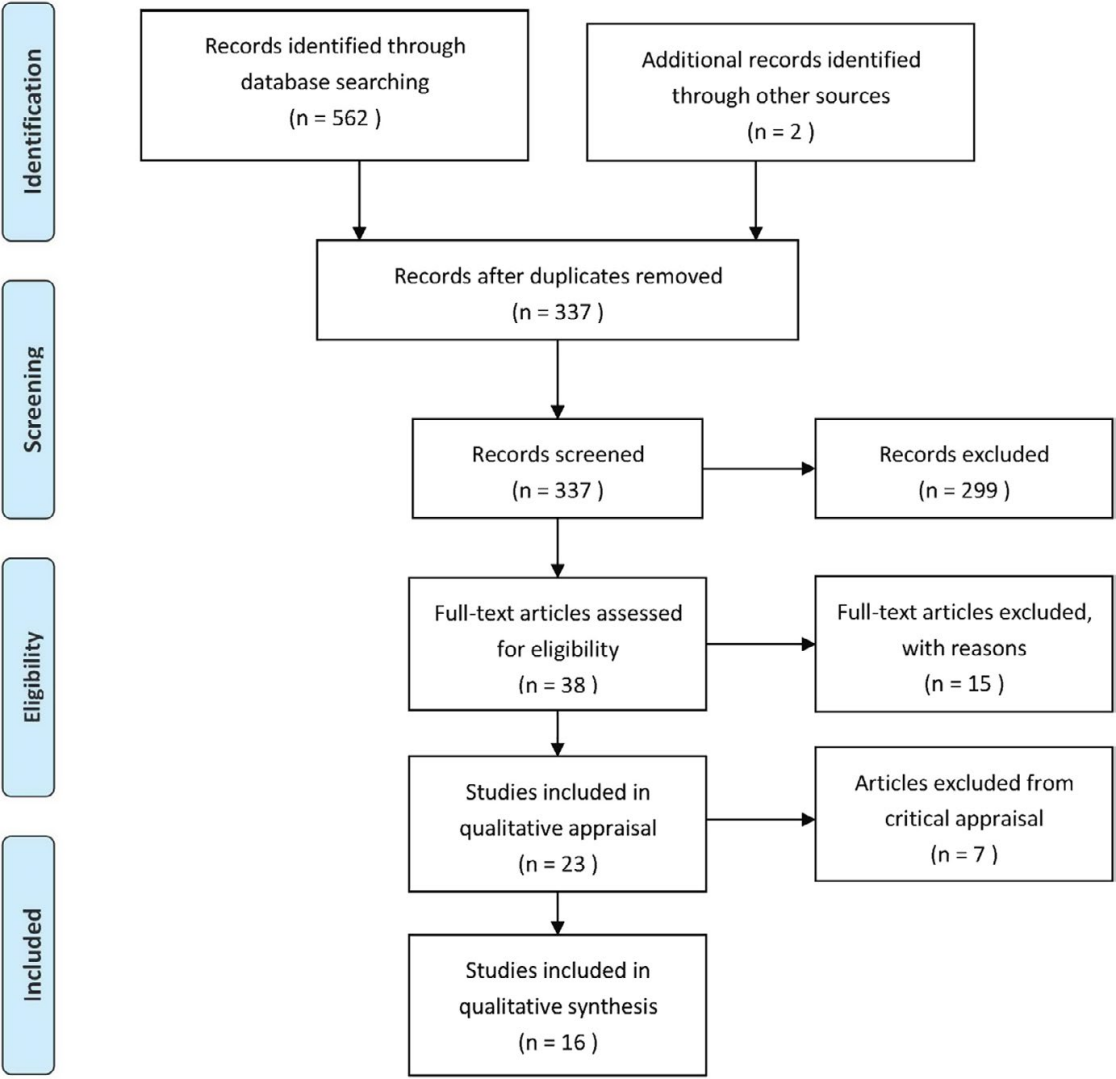
PRISMA flow diagram of study selection and inclusion process.*Source:*Moher et al. (2009) [Colour figure can be viewed at wileyonlinelibrary.com]

As shown in Figure 1, a total of 564 studies (562 from selected databases and two from other sources) were collected and then imported into EndNote (Clarivate Analytics, PA, USA) bibliographic software. After the removal of 227 duplicates, a total of 337 studies were screen for eligibility and 299 of them irrelevant to the topic were excluded. The remaining 38 potentially relevant articles were subject to further detailed assessment of eligibility by reviewing the full text, and 23 papers that met the inclusion criteria were selected for critical appraisal. The 23 papers were then critically appraised by two reviewers, and 16 of them were agreed be included in the review. The other seven papers were excluded because they were not rated highly enough in methodological quality for inclusion in the review (Appendix S3).

### 3.2 Methodological quality

The included papers were critically appraised using the JBI Qualitative Critical Appraisal Checklist (Table 1). All the studies were rated positively in research methodology (criteria 2, 3, 4, 5) and were adequate representation of participants voices (criteria 8). Meanwhile, all the studies were also rated positively incongruity between the stated philosophical perspective and the research methodology, and the conclusions were drawn rationally. Nine papers stated the influence of the researcher on the research. Overall, the methodology quality of included studies was verified.

### 3.3 Description of included studies

A total of 16 research studies were included in the review. Among these 16 papers, 12 were from the United States and the others were from Australia, Korea, Sweden and the UK, respectively. As for the setting of research, 13 studies focused on war, one study included both war and non‐war military operations, and the other two studies did not state the type of deployment.

### 3.4 Meta‐synthesis of qualitative data

Thirty findings were extracted from the 16 studies included in the synthesis (Table 2). All 30 findings were rated as either “Credible” or “Unequivocal” (Appendix S4). The 30 findings were aggregated into 12 categories, which were subsequently synthesized into five synthesized findings.

**Table 2:** Results of meta‐synthesis

Finding	Category	Synthesized finding	Phase
Preparing for an uncontrolled environment	Mental, physical and professional preparation	Preparing and sharing experience are the key coping strategies	Preparation
Preparing to deal with anxiety			
Understanding the mission			
Training			
Talking about your experiences	Share experience by telling or writing down		
Advice about journaling			
A changing world	Uncertain physical environment	Transition from the civilian care to emergency situations	Deployment
Sacrificing physical safety			
Enduring confusion	Complex psychological environment		
Being discriminated against as female			
Facing moral and ethical dilemmas			
Level of care	Different professional environment and practice scope		
Dealing with supply issues			
Cultural divide			
Team working	Teamwork and bonding	Teamwork contributing to bonding and the growing role of nursing in medical team	
Kinship and bonding			
The change of social view towards nursing profession	Increasing professionalization of nursing		
Take important roles at managerial and political decision‐making			
Many roles they perform			
Being devoted to duty	Committed to duty	Devoting to nursing duty achieves growth	
Giving of oneself			
Professional growth: expanding my skills	Personal and professional development		
Growing as leaders			
Achieving and being rewarded			
Command support: no one Cared	Family/command/social support matters during reintegration	Reintegration is not easy and external support matters	Reintegration
Family and social networks: support versus lack of support			
PTSD symptoms	Psychological concern should be paid attention		
Sorting it out: getting help			
Homecoming: A Mixed Reception	Transition from deployment to a new normal life		
Reintegration: a new normal			

#### 3.4.1 Preparation phase

##### Synthesized finding 1: Preparing and sharing experience are the key coping strategies

This synthesized finding is underpinned by six extracted findings and is subdivided into two categories: “Mental, physical and professional preparation” and “Sharing experience by telling or writing down”. This summarizes that an adequate preparation is important for nurses to make through all kinds of challenges during deployment.

The first category “Mental, physical and professional preparation” is developed from four extracted findings: “Preparing for an uncontrolled environment”, “Preparing to deal with anxiety”, “Understanding the mission” and “Training”. These findings state that nurse should be fully prepared for the transition from civilian care to a changing world in mental, physical and professional aspects.

The further you get in the preparatory training, the clearer the picture becomes of what it is like down there. The various elements become less strange and you feel that you have mastered them. (Ekfeldt et al., 2015, p5).

The second category “Sharing experience by telling or writing down” is developed from two extracted findings: “Talking about your experiences” and “Advice about journaling”, which reflects that it is necessary for nurses to speak out their stressful experiences to promote mental health.

My journal helped me remember people, what I was thinking in those days, and how I reacted to situations. I look back on it now, and it was very useful in helping me put Vietnam in perspective. (Scannell‐Desch, 2005, p603).

#### 3.4.2 Deployment phase

##### Synthesized finding 2: Transition from the civilian care to emergency situations

This synthesized finding is underpinned by eight extracted findings and is subdivided into three categories: “Uncertain physical environment”, “Complex psychological environment” and “Different professional environment and practice scope”. Overall, nurses during deployment are living and working in the brand new physical, psychological and professional environment distinguished from their daily working environment.

The first category “Uncertain physical environment” is developed from two extracted findings: “A changing world” and “Sacrificing physical safety”, which describes the physical environment during deployment. Nurses described the deployment environment, especially combat zone, to be chaos and danger, accompanied with extreme weather condition.

You were almost detached from the whole situation, because we were 100ks back off the frontline, so apart from the alarms going off for the scud attacks that was about the only thing that really made you think ‘Oh, maybe I could be in danger’. (Griffiths & Jasper, 2008, p95).

The second category “Complex psychological environment” is developed from a total of three findings: “Enduring confusion”, “Being discriminated against as female” and “Facing moral and ethical dilemmas”, which describes how nurses struggle to find the balance in their dual roles as nurse and soldier when working in military nursing context. Some nurses went through confusion, compassion fatigue, anxious and felt discriminated as female in the male‐dominated military. Also, nurses suffered ethical conflicts when they could not provide more care to patients because of the limited resource.

Sometimes I look back and wonder if these guys have cursed us for saving them because they have handicaps, like the spinal cord injuries or the one that lost several limbs. It bothered me that some of the policies and politics would not allow the fighting to be conducted in a way it was supposed to be. (Scannell‐Desch, 1996, p123).

The third category “Different professional environment and practice scope” is developed from three extracted findings: “Level of care”, “Dealing with supply issues” and “Cultural divide”, which reflects different practice scope from civilian care in hospital. Military nurses described that they have provided care to patients of all ages with multi‐cultural background, and the medical service they provided was centred on trauma care. Meanwhile, they might face limited supply and have to learn improvisation.

Horrific injuries where you got flesh hanging all over the place, big bullet holes. I’ve never seen anybody shot before and just seeing a bullet go in one side where you get this quarter size injury hole and then you cut and see the back of somebody's calf, for instance, and there's this four or five inch cross exit wound…It's just different; you have to be prepared for all different types of injury. (Agazio, 2010, p171).

##### Synthesized finding 3: Teamwork contributing to bonding and the growing role of nursing in medical team

This synthesized finding is underpinned by five extracted findings and is subdivided into two categories: “Teamwork and bonding” and “Increasing professionalization of nursing”. Nurses, who shoulder many responsibilities in the health care team, contribute to mutual corporation and teamwork.

The first category “Teamwork and bonding” is developed from two extracted findings: “Team working” and “Kinship and bonding”, which reflects the importance of teamwork and how individuals in a team bond with each other.

We didn't pay attention to the label of people in the medical team … Our relationships were entirely based on mutual cooperation; and my colleagues and I were as two partners. (Peyrovi et al., 2015, p21).

The second category “Increasing professionalization of nursing” is developed from three extracted findings: “The change of social view towards nursing profession”, “Taking important roles at managerial and political decision‐making” and “Many roles they perform”. Together, these findings summarize the growing role and professionalization of nursing in a medical team.

We defined a managerial process for reducing complications in casualties. This process was the process [of transfer] from the front line of the battle to the third line. In fact, we designated a care management process that was very successful. (Peyrovi et al., 2015, p18).

##### Synthesized finding 4: Devoting to nursing duty achieves growth

This synthesized finding is underpinned by five extracted findings and is subdivided into two categories: “Committed to duty” and “Personal and professional development”. Overall, many nurses have reported how they were committed to nursing duty and what they have learned and grown during deployment, both personally and professionally.

The first category “Committed to duty” is developed from two extracted findings: “Being devoted to duty” and “Giving of oneself”, which describes the spirit of being a military nurse and rescuing the injured.

Whenever I felt fear, I told myself not to worry about anything other than thinking about how best to do my work on the battlefield. As an officer, I thought it would be an honor if I died there. (Han, 2019, p3).

The second category “Personal and professional development” is developed from three extracted findings: “Professional growth: expanding my skills”, “Growing as leaders” and “Achieving and being rewarded”. These findings state that nurses gain personal and professional growth such as a sense of achievement, expanding skills and learning leadership.

I enjoy the opportunity to share my knowledge and experience and have actually conducted in services for nurses. For me, that is rewarding—to have the chance to be a teacher and trainer. (Goodman et al., 2013, p1012).

#### 3.4.3 Reintegration phase

##### Synthesized finding 5: Reintegration is not easy and external support matters

This synthesized finding is underpinned by six extracted findings and is subdivided into three categories: “Family/command/social support matters during reintegration”, “Psychological concern should be paid attention” and “Transition from deployment to a new normal life”. In summary, transition from deployment to routine life is not easy, and is often overlooked.

The first category “Family/command/social support matters during reintegration” is developed from two extracted findings: “Command support: no one cared” and “Family and social networks: support versus lack of support”, which reflects the importance of external support during reintegration.

My colleagues were supportive. They wanted to know what I did, how it went, and what I saw. It was helpful to speak of it. I think the more people hear about our experiences, the more they can understand why some of us come back with PTSD [posttraumatic stress disorder] or compassion fatigue. (Doherty & Scannell‐Desch, 2015, p32).

The second category “Psychological concern should be paid attention” is developed from two extracted findings: “PTSD symptom” and “Sorting it out: getting help”. Nurses usually have to bear heavy workload during deployment, especially in war zone, where they need to care a large number of severe patients. Some nurses are reported to have symptom of post‐traumatic stress disorder (PTSD), and thus, psychological support is critical for their mental health.

When you come home and try to readjust that's the difficult part because the stuff that we've seen, the stress that you face when you are put in that position up in the plane or the helicopter in the middle of the night—all that stuff—you just can't unsee that. (Rivers et al., 2017, p248).

The third category “Transit from deployment to a new normal life” is developed from two extracted findings: “Homecoming: A mixed reception” and “Reintegration: a new normal”, which describes the phenomenon that nurses are willing to go back home while being stressed facing family affair and duty at work. Eventually, they are likely to develop a different daily life routine.

When I come home from deployments, the key is not trying to do too much too soon. I learned that from multiple deployments. I learned that it was not always good to go right home to family. If you can take a week to decompress and be by yourself, do it! I must say I have never been a person with a temper, but it has probably gotten a little shorter after having three deployments. (Doherty & Scannell‐Desch, 2015, p33).

## 4 DISCUSSION

This systematic review concluded military nurses’ experiences of deployment focusing on the three phases of deployment cycle. The selected 16 papers in the meta‐synthesis resulted in 30 findings that were summarized into 12 categories and further concluded as five synthesized findings.

Evidences have suggested that nurses experienced a range of psychological reactions and felt unprepared during deployment. Lal and Spence (2016) and Rivers and Gordon (2017) reported that nurses felt anxious, worrying, angry and lonely. Meanwhile, it was reported that nurses are likely to insufficiently prepare and feel unconfident during deployment (Labrague et al., 2018). Therefore, a full preparation plays an important role for military nurses to successfully complete their duties. Professionally, military nurses should master the nursing skills such as emergency care, intensive care, trauma care and infectious disease care in respond to a variety of emergency situations during deployment. Physically, uncontrolled environment, such as extreme weather condition and uncertain workplace, should be considered in the preparation protocol. Personally, team and family supports are necessary in order to relief nurses’ anxiety for the deployment. Mentally, nurses should acknowledge and understand the difficulty of the mission and thus be ready for all kinds of challenges during deployment.

This review indicated the growing role of nursing with increasing professionalization. Nurses actively participate in services to promote health, to prevent illness, to restore health and to alleviate suffering according to the code of ICN; demonstrate their capabilities for crisis management; and take important roles in decision‐making (Peyrovi et al., 2015). Indeed, nurses shoulder many roles in disaster preparedness and response, for instance, as leaders, educators and researchers (Veenema et al., 2016). This review also supported that nurses experienced personal and professional development while demonstrating the capacities of nursing team. Furthermore, it was clear that the deployment is a good chance for nurses to expand skills and gain experiences.

This review emphasized the importance of management and support to nurses during reintegration, which was neglected to some extent. First, the adaption time of reintegration depends on the challenge military nurses encountered during deployment and is expected to be 4–8 weeks. However, in some extreme cases, the reintegration has become a lifelong process for nurses (Rivers et al., 2013). Second, progressive interventions such as post operational stress management, command support and family support should be tailored for nurses and introduced during the process of reintegration (Finnegan et al., 2016). Therefore, the collaboration between strength at individual, interpersonal, organisational and societal levels is essential to help them back to normal life. Additionally, due to the fact that many nurses are suffering from PTSD or PTSD‐like symptoms during the reintegration, psychological consultation should be applicated after deployment (Rivers et al., 2017).

One limitation of this review is that the type of deployment among most of the included studies is war. It is important to note that nurses might have different experiences during deployment to non‐war operations such as disasters and public emergencies, and thus, the responding strategies might be different. Other limitations include language barrier and distributed limited files. We tried expanding search strategies for more relevant published studies. However, due to the restricted distribution of relevant military files and language barrier, the resources we could get access to were limited.

## 5 CONCLUSION

This systematic review aimed to identify, critically appraise and synthesize qualitative evidences on the preparation, the deployment and the reintegration experiences of military nurses. The conclusion drawn from these studies is that it is difficult for military nurses to adapt the transition from civilian care to an uncertain environment while the reintegration from structured deployment environment to informal family environment, and thus, they will be more likely to survive the deployment via being well prepared, having support, as well as achieving professional and personal growth. Since nurses shoulder the responsibility of providing quality health care services under any circumstance, we should explore how to maintain a competent, stable and ready nursing team while mitigating the negative effect of deployment on nurses in the future research.

## 6 IMPLICATIONS FOR NURSING MANAGEMENT

Nurse managers should consider the competency and readiness of a nursing team when responding to disasters and public emergencies. Regular and formal training can boost the confidence and the capacity of nurses. Managers should also acknowledge that the deployment experience is a challenge for nurses, and they should provide sufficient supports in each stage of deployment. Meanwhile, nursing association is a key stakeholder in developing policies to provide better working environment and more support to nurses. Overall, the synthesis findings can be used to develop the competency building and training protocol of the preparation, the deployment and the reintegration and offer implications for nurse mangers on how to sustain a competent and ready nursing team.

## ETHICAL APPROVAL

Ethical approval was not required for this paper.

## Supporting information

Appendix S1

Appendix S2

Appendix S3

Appendix S4
