# Supplementary material for: Deployment experiences of military nurses: A systematic review and qualitative meta‐synthesis
Source: J Nurs Manag. 2020 Nov 20;29(5):869–77. doi: 10.1111/jonm.13201 (PMC8359314; doi:10.1111/jonm.13201)
Supplement: Supplementary file 1 — Appendix S1 [file JONM-29-869-s003.docx]

**Appendix I: Search strategies**

Search strategies used for finding qualitative research articles about deployment experiences of military nurses. Number of retrieved articles is given in the right-hand column.

**PubMed**

| S1 | nurs*[Mesh, ti, ab] | 501,959 |
| --- | --- | --- |
| S2 | “military” OR “army” OR “air force” OR “navy” OR “warrior” [Mesh, ti, ab] | 68,544 |
| S3 | “deploy*” OR “reintegration” OR “homecoming” OR “war*” OR “humanitarian” OR “disaster” [Mesh, ti, ab] | 137,647 |
| S4 | “experience*” OR “qualitative” OR “interview*” OR “focus group*” [Mesh, ti, ab] | 1,535,258 |
| S5 | S1 AND S2 AND S3 AND S4 | 127 |

**CINAHL**

| S1 | nurs*[AB] | 338,519 |
| --- | --- | --- |
| S2 | “military” OR “army” OR “air force” OR “navy” OR “warrior” [AB] | 18,645 |
| S3 | “deploy*” OR “reintegration” OR “homecoming” OR “war*” OR “humanitarian” OR “disaster” [AB] | 30,485 |
| S4 | “experience*” OR “qualitative” OR “interview*” OR “focus group*” [AB] | 542,816 |
| S5 | S1 AND S2 AND S3 AND S4 | 240 |

**EMBASE**

| S1 | nurs*[ ti, ab] | 541238 |
| --- | --- | --- |
| S2 | “military” OR “army” OR “air force” OR “navy” OR “warrior” [ ti, ab] | 75395 |
| S3 | “deploy*” OR “reintegration” OR “homecoming” OR “war*” OR “humanitarian” OR “disaster” [ ti, ab] | 708585 |
| S4 | “experience*” OR “qualitative” OR “interview*” OR “focus group*” [ ti, ab] | 206464764 |
| S5 | S1 AND S2 AND S3 AND S4 | 127 |

**PsycINFO**

| S1 | nurs*[AB] | 25930 |
| --- | --- | --- |
| S2 | “military” OR “army” OR “air force” OR “navy” OR “warrior” [AB] | 20548 |
| S3 | “deploy*” OR “reintegration” OR “homecoming” OR “war*” OR “humanitarian” OR “disaster” [AB] | 138521 |
| S4 | “experience*” OR “qualitative” OR “interview*” OR “focus group*” [AB] | 337206 |
| S5 | S1 AND S2 AND S3 AND S4 | 63 |

**Cochrane Library**

| S1 | nurs*[ ti, ab] | 614 |
| --- | --- | --- |
| S2 | “military” OR “army” OR “air force” OR “navy” OR “warrior” [ ti, ab] | 31 |
| S3 | “deploy*” OR “reintegration” OR “homecoming” OR “war*” OR “humanitarian” OR “disaster” [ ti, ab] | 33 |
| S4 | “experience*” OR “qualitative” OR “interview*” OR “focus group*” [ ti, ab] | 1263 |
| S5 | S1 AND S2 AND S3 AND S4 | 5 |

TOTAL FOUND: 562
