# Supplementary material for: Deployment experiences of military nurses: A systematic review and qualitative meta‐synthesis
Source: J Nurs Manag. 2020 Nov 20;29(5):869–77. doi: 10.1111/jonm.13201 (PMC8359314; doi:10.1111/jonm.13201)
Supplement: Supplementary file 2 — Appendix S2 [file JONM-29-869-s002.docx]

**Appendix II: Characteristics of included studies**

| **Author (year), country** | **Methodology** | **Methods** | **Phenomena of Interest** | **Setting** | **Demographics of Participants** | **Data analysis** | **Conclusion** |
| --- | --- | --- | --- | --- | --- | --- | --- |
| Han (2019),  Korea | Hermeneutic phenomenological approach | Face-to-face interview | To explore the essence of lived experiences among Korean female nursing officers | Vietnam War | 14 Korean female nursing officers | Thematic  analysis | This study acquired valuable data on nursing history and useful information on the psychological, physical, and environmental difficulties that could be faced by female nurses working in conflicts, wars, and disaster situations. |
| Conlon, Wiechula & Garlick (2019),  Australia | Hermeneutic phenomenological approach | Teleconference interview | To gain an understanding of the lived experiences of nursing officers when working as a member of a military trauma team | - | 6 Military Nursing Officers | Thematic analysis | This study has provided an insight into the experiences of six Australian military nurses working in trauma teams experiences that were rarely given voice outside the military. Central to this experience is the need to have these stories told. |
| Rivers et al. (2017) , USA | Mixed-methods analytic approach | One-on-one interview | To better understand the post-deployment behavior health symptoms and reintegration experienced by military nurses who provided en route care | Operation  Enduring Freedom/Operation Iraqi Freedom | 22 military en route care nurses | Thematic analysis | Combined results indicated en route care nurses encountered difficulties when attempting to return to predeployment roles; behavioral health problems mirrored those of combat warriors. Interventions to assist post-deployment reintegration of en route care nurses should be conducted at the peer, leader, and health care provider levels. |
| Peyrovi et al. (2015), USA | Oral history research method | Face-to-face interview | To analyse the history of the wartime experience of Iranian nurses in Iran-Iraq War | Iraq-Iran War | 13 Iranian military nurses | Thematic analysis | Nursing in Iran at wartime has a difficult path to development. There are powerful implications for clinical practice. It is recommended to continue collection, archiving and analysing the wartime experiences of Iranian nurses. |
| Elliott (2015), USA | Narrative inquiry | Interview | To describe the military nurses’ post-deployment experiences and their meaning | war | 10 military nurses | Thematic analysis | It is critical for military nurses and leaders, healthcare providers, nursing administration/educators, as well as nurses who work alongside military nurses, both in the USA and in other countries, to have a better understanding of the meaning of the deployment experience so they may provide support to these nurses during the post-deployment phase. |
| Ekfeldt, Ekfeldt & Nyström (2015), Sweden | Qualitative design | Interview | To describe military nurses’ experiences of preparations for a mission, including factors that contribute to their being meaningful. | Afghanistan war | 8 Swedish military nurses | Qualitative content analysis | Organized preparation for care in a combat environment should stimulate realistic reflection without entrapping one in negative thinking. |
| Doherty &Scannell-Desch (2015), USA | Phenomenological method | Interview | To describe reintegration experiences of U.S. military nurses returning from deployments in the Iraq and Afghanistan wars. | Iraq or Afghanistan war | 35 U.S. Army, Navy, or Air Force nurses | Colaizzi’s analysis | These nurses struggled with reintegration on many levels and with different factions, including family, coworkers, friends, and their communities. |
| Rivers et al., (2013), USA | Phenomenological method | Interview | To understand U.S. Army nurses’ reintegration and homecoming experiences after deployment to Iraq or Afghanistan | Iraq or Afghanistan war | 22 U.S. Army active duty nurses | Thematic analysis | Nurses in this study felt that the current reintegration process was not meeting their needs for a smoother homecoming; new or improved interventions to assist redeploying nurses with the transition to a noncombat environment would be beneficial. |
| Goodman et al., (2013), USA | Phenomenological inquiry | Focus group | To understand military nurses’ experiences of care  for Iraqi patients. | Iraq war | 15 U.S. active duty Army nurses | Thematic analysis | The themes support existing research and extend information about care of host nation patients adding depth and breadth to specific content areas. These nurses  developed situated knowledge needed for particular challenges and experienced personal and professional growth. |
| Scannell-Desch & Doherty (2010), USA | Phenomenological method | Interview | To describe the lived experience of U.S.military nurses who served in Iraq or Afghanistan during the war years 2003 to 2009, and life after returning from war. | Iraq or Afghanistan war | 37 U.S. Army, Navy, or Air Force nurses | Colaizzi’s analysis | Nursing in war is a unique experience regardless of education, preparation and training. There are a myriad of variables that enter into the experience and effect outcomes, both personal and professional. |
| Agazio (2010),  USA | Descriptive exploratory design | Interview | To provide a more complete description of Army nursing practice challenges in MOOTW as compared with wartime operations as more recently experienced in Operation Enduring Freedom and Operation Iraqi Freedom. | War and non-war military operation in Bosnia, Honduras, Iraq, Afghanistan and other worldwide locations | 75 U.S. Army Nurse Corps officers | Qualitative data analysis | Nurses recounted challenges and adjustments made to deliver high quality patient care to soldiers and civilian casualties especially for multi-level traumatic injuries. Specialized skill sets and personal adaptation were necessary for practice under austere conditions in these environments. By understanding the practice of nursing in MOOTW and wartime, nurses can best prepare and train to effectively function and care for patients in these challenging settings. |
| Rushton, Scott & Callister (2008),  USA | Qualitative design | Audio-taped interview | To permanently archive their accounts, to generate themes that elucidate their nursing experiences, and to honor nurses that have served by sharing their stories. | Persian Gulf Wars | 10 U.S. Navy Nurse and 1 Air Force Nurse | Thematic analysis | Military nurses’ personal stories demonstrated the importance of being engaged in making meaningful professional and historical contributions. These nurses displayed professional commitment and hardiness in the face of difficult life circumstances, saying, “We did what we had to do.” |
| Griffiths &Jasper (2008), UK | Grounded theory design | In-depth interview and focus group | To explore the nature of military nursing in an environment of war, and to identify the actual or potential effect this had on the nursing  role in this unique environment | war | 24 UK Royal Navy, Army  and Royal Air Force nurses | Constant  comparative analysis | The symbiotic relationship of carer and warrior arises as a consequence of strategies used by military nurses to embrace their dual role. Further research is needed to explore the essence of the caring role within a conflict zone from military and civilian perspectives. |
| Scannell-Desch(2005), USA | Phenomenological approach | Interview | To describe guidance for nurses today from the lessons  learned by nurses who served in the Vietnam War. | Vietnam war | 24 U.S. Navy, Army and Air Force nurses | Thematic analysis | Nurses need to take a pro-active role in preparing themselves for deployment to a war zone, and that institutional training for war needs to be intensive and realistic. The environmental, cultural, technological, clinical and psychosocial demands of war nursing need to be comprehensively addressed before nurses deploy to a war. |
| Cox (2005), USA | Phenomenological inquiry | Tape-recorded face-to-face interview | To describe the experience of shipboard nursing on aircraft carriers. | - | 12 Navy nurses | Thematic analysis | The findings have provided valuable insight into nursing practice in a service-unique environment and will also assist Navy Nurse Corps leaders with making appropriate assignments for nurses seeking a job on a  ship. |
| Scannell-Desch(1996), USA | Phenomenological inquiry | In-depth interview | To explore common components of the lived experience of women military nurses who served in Vietnam and common elements of life after returning from Vietnam | Vietnam war | 24 U.S. Navy, Army and Air Force nurses | Thematic analysis | The Vietnam War continues to have an effect on the lives of the nurses who served there. They balance their personal and professional growth gleaned from this experience with the physical and emotional stresses experienced during the war and since the war. |
