# Supplementary material for: Deployment experiences of military nurses: A systematic review and qualitative meta‐synthesis
Source: J Nurs Manag. 2020 Nov 20;29(5):869–77. doi: 10.1111/jonm.13201 (PMC8359314; doi:10.1111/jonm.13201)
Supplement: Supplementary file 3 — Appendix S3 [file JONM-29-869-s004.docx]

**Appendix III: Excluded studies**

Stanton, M., Parker, M. W., McDougall, G., & Eyer, J. C. (2017). Reintegration Issues of Military Nurses: A Focus Group Approach. Best practices in mental health, 13(1), 1-19.

**Reason for exclusion:** no evidence of ethics approval or data that represents participant’s voices.

Almonte A. L. (2007). Navy nurses’ experiences during Operation Unified Assistance aboard the USNS MERCY: a grounded theory study [Ph.D., University of San Diego]. In Navy Nurses’ Experiences During Operation Unified Assistance Aboard the USNS MERCY: A Grounded Theory Study.

**Reason for exclusion:** no evidence of ethics approval or lack of data that represents participant’s voices in some themes.

Almonte A. L. (2009). Humanitarian nursing challenges: a grounded theory study. Military medicine, 174(5), 479-485. https://doi.org/10.7205/milmed-d-01-7908

**Reason for exclusion:** no evidence of ethics approval or lack of data that represents participant’s voices in some themes or themes are not quite clear.

O’Neill TM. (2003). “I wanted to do something for the country”: experiences of military nurses in World War II [Ph.D., University of Miami]. In I Wanted to Do Something for the Country: Experiences of Military Nurses in World War Ii.

**Reason for exclusion:** lack of data that represents participant’s voices in some themes

LeVasseur JJ. (2003). The proving grounds: combat nursing in Vietnam. Nursing Outlook, 51(1), 31–36.

**Reason for exclusion:** evidence of ethics approval or lack of data that represents participant’s voices in some themes.

Turner DMH. (1998). The experience of chief nurses in military operations other than war [Ph.D., University of Minnesota]. In Experience of Chief Nurses in Military Operations Other Than War (p. 189 p).

**Reason for exclusion:** interpretive clusters have date that represents participant’s voices, however themes are developed from around five interpretive clusters, thus themes are complex and big to explain and understand.

Stanton, M. P., Dittmar, S. S., Jezewski, M. A., & Dickerson, S. S. (1996). Shared experiences and meanings of military nurse veterans. Image--the journal of nursing scholarship, 28(4), 343–347. https://doi.org/10.1111/j.1547-5069.1996.tb00385.x

**Reason for exclusion:** lack of data that represents participant’s voices in some themes.
