# Supplementary material for: Deployment experiences of military nurses: A systematic review and qualitative meta‐synthesis
Source: J Nurs Manag. 2020 Nov 20;29(5):869–77. doi: 10.1111/jonm.13201 (PMC8359314; doi:10.1111/jonm.13201)
Supplement: Supplementary file 4 — Appendix S4 [file JONM-29-869-s001.docx]

**Appendix IV: Study findings and illustrations**

Han J. J. (2019). The Lived Experience of Korean Female Military Nursing Officers During the Vietnam War. Journal of transcultural nursing : official journal of the Transcultural Nursing Society, 30(5), 471–477. https://doi.org/10.1177/1043659618818713

| **Finding** | **Illustrations from study** | **Evidence** |
| --- | --- | --- |
| Enduring confusion | After nine days on the ship, I finally could see the land, far away. How beautiful it might be! The sea breeze blowing in my face was as soft as a silk scarf. But in the middle of that night, suddenly it became as bright as day due to the bursting of star shells. Everyone shouted and jumped out of the tent. From then on, we wore helmets even during sleep to protect us from brain injuries and to survive. (p3) | Credible |
| Being devoted to duty | Even when I was going to the stream to wash my face, I was afraid that the Vietcong would be hiding in the trees and would shoot me. Whenever I felt fear, I told myself not to worry about anything other than thinking about how best to do my work on the battlefield. As an officer, I thought it would be an honor if I died there. (p3) | Credible |
| Establishing deep comradeship | I worried that the wounded soldiers might be depressed because of their disabilities, so after finishing my duty, and even on weekends, I stayed in hospital and played the guitar to console them in their pain. (p4) | Credible |
| Realizing the dark side of war | A local female nurse aid was pregnant and the due date was coming closer. She said that the father of the baby was a Korean military medic who returned home and never contacted her. (p4) | Credible |
| Being discriminated against as female | In contrast to the other department head officers, the chief nursing officer was not assigned a private military jeep. I explained to the troop commander the need for it to supervise the night shift nurses, who worked in the hospital, a long way from my quarters. Soon, he supplied a jeep for me to the hospital. However, the logistics officer kept it from me and tried to give it to another male officer. (p4) | Unequivocal |
| Achieving and being rewarded | One of the evacuated soldiers said that they told each other if they saw a female nursing officer at the moment of opening their eyes, they were alive and did not have to worry. I was very thankful and felt a greater responsibility and sense of reward. (p4) | Unequivocal |
| Growing as leaders | The ROK military temporarily placed the remains of the war dead in Vietnam in the charnel house, sending them to Korea every month after a funeral ceremony. We realized that many Koreans were returning home in ashes. On the way to the hospital, so many thoughts would flood in. What was the war for? Why did our people have to die in a foreign country? I guess you can say that I grew through such a process. In retrospect, nursing officers seem to have had many opportunities to think and grow on the battlefield. (p5) | Unequivocal |

Conlon, L., Wiechula, R., & Garlick, A. (2019). Hermeneutic Phenomenological Study of Military Nursing Officers. Nursing research, 68(4), 267-274. https://doi.org/10.1097/NNR.0000000000000342

| **Finding** | **Illustrations from study** | **Evidence** |
| --- | --- | --- |
| Telling their stories | I know you’re [the researcher] military, so we subconsciously talk to people we know will understand. (p268) | Credible |
| The Uniqueness of Being a Military Nurse | We were in a war zone, [it was] very dangerous. We had up to five to 10 contacts [from the enemy] a day and it was pretty hairy. (p269) | Credible |
| The Many Roles They Perform P | I see myself as a mentor and or facilitator for the junior medics and other nurses in the team. (p269) | Unequivocal |
| Being a Valued Member of the Team | These girls are my heroes…the nurses… were technically competent and I was as proud as punch that when we looked at things we wanted to do, that the Americans wanted Australians on their team. (p269) | Credible |
| The Physical Environment | It’s a different world. You can’t compare the two. (p269) | Unequivocal |
| The Psychosocial Environment | …the guy that gets shot, the guy who gets blown up by an IED [improvised explosive device], he is a guy that you used to live with…worked and played with, and all the rest of it. (p270) | Credible |
| Training | We had seen some stuff that no one had ever prepared for…there was nothing that could really prepare us for what we saw. (p270) | Credible |
| Preparing for an Uncontrolled Environment | Nothing ever prepares you for that emotional response and when the adrenaline kicks in…. And while nothing prepares you for that, once that happens, your military training just kicks in. (p270) | Credible |
| Training Without the Team | What was lacking was an intimate knowledge of each other’s strengths and weaknesses… this was something that could be really developed by being trained as a team.(p270) | Unequivocal |
| The Team | Short answer is, no; we didn’t. We didn’t have formal teams…. It was rotated because the nurses worked shift work and it was whoever happened to be on that particular shift. (p271) | Credible |
| Knowing Your Place in the Team | I think the most poignant thing in the resus, it has nothing to do with drugs, but putting in an IDC [indwelling catheter] with a suspected fractured pelvis and I was putting in the IDC and said to the surgeon, “I think I can’t go any further. I think there’s some trauma there.” And he told me to keep pushing and I wouldn’t. So, I withdrew the catheter and there were all these big clots came out of his [the patient’s] urethra. And I thought, looking at the surgeon, I probably give him the look of, see there you go. I was a Flying Officer, so I’m surprised I never got charged! (p271) | Credible |
| Leadership | The composition of each of the teams was a nurse as the leader. There were three nurses and about six medics. The medics were split across the nurses, and we had two doctors. (p271) | Unequivocal |

Rivers, F. M., Dukes, S., Hatzfeld, J., Yoder, L. H., Gordon, S., & Simmons, A. (2017). Understanding Post-Deployment Reintegration Concerns Among En Route Care Nurses: A Mixed-Methods Approach. Military medicine, 182(S1), 243–250. https://doi.org/10.7205/MILMED-D-16-00209

| **Finding** | **Illustrations from study** | **Evidence** |
| --- | --- | --- |
| Career challenges, “leadership matters” | I was anticipating coming back in December, [but] I was sent home in November ... they gave me a four-day pass for Thanksgiving. I came back and just started working ... they gave me Christmas off and I worked until I took leave in January. (p246) | Credible |
| Social difficulties, “I Don’t Fit In” | [Trying to fit in] and how I handle things now that I’ve come back is difficult. It’s very hard for me to hear people complain ... I’m not handling that part well. (p247) | Unequivocal |
| Intimate relationship problems, “here is my suffering” | My family and friends ... I just didn’t want to talk to them about it because a lot of times I would think that they wouldn’t understand. (p247) | Unequivocal |
| Health concerns, “Here is my suffering” | I spent six weeks in an intensive outpatient group for PTSD. I’ve seen a psychiatrist monthly and a psychologist at least two times a month. I’ve been in the step-down PTSD group twice a week for the better part of the year now. I’m still dealing with the issues. I take more medication than anybody should have to take. (p247) | Unequivocal |
| Concerns about deployment, “I do not fit in | It’s almost like things here can be so trivial sometimes after what you see down range.” “I realized when I got back; I was no longer tolerant of the little stuff. (p248) | Credible |
| PTSD symptoms, “terror of war—you can’t unsee that” | When you come home and try to readjust that’s the difficult part because the stuff that we’ve seen, the stress that you face when you are put in that position up in the plane or the helicopter in the middle of the night—all that stuff—you just can’t unsee that. (p248) | Unequivocal |

Peyrovi, H., Parsa-Yekta, Z., Vosoughi, M. B., Fathyian, N., & Ghadirian, F. (2015). From margins to centre: an oral history of the wartime experience of Iranian nurses in the Iran-Iraq War, 1980-1988. Contemporary nurse, 50(1), 14–25. https://doi.org/10.1080/10376178.2015.1010258

| **Finding** | **Illustrations from study** | **Evidence** |
| --- | --- | --- |
| The change of social view towards nursing profession | Nursing at wartime could attract all the community. In fact, nursing could represent itself, and this happening was what we were looking for. (p17) | Credible |
| Demonstration of nursing capabilities for crisis management | Believe it or not, we could triage 50 to 60 wounded patients within two hours at the front line emergency departments, and then they would transfer to the wards quickly. (p18) | Credible |
| Take important roles at managerial and political decision making | We defined a managerial process for reducing complications in casualties. This process was the process [of transfer] from the front line of the battle to the third line. In fact, we designated a care management process that was very successful. (p18) | Unequivocal |
| Frontline care | In Khat-e-Moghadam [frontline], we faced high risk [situations]. There were bullets and shrapnel. We excavated a trench there and transferred victims into it. It was called Post-e-Emdad. At Post-e-Emdad, we did initial interventions to save a life. Then, we moved injuries back to the emergency department. Emergency departments were equipped with about 10 beds. In emergency departments, nurses tried to more stabilize injuries conditions. (p18) | Unequivocal |
| The second line care | The maximum distance of Sahraee hospitals from Khat-e-Moghadam [frontline] was usually 40–50 kilometers. These hospitals were very equipped. Approximately, since 1981 or 1982 we have Sahraee hospitals … , for example, Fatemeh-Zahra hospital, it was a field hospital with 20 operating rooms that provided advanced care to victims. (p19) | Unequivocal |
| Third line of care | Nurses had an effective role in care management of injuries at the center of provinces and non-war cities. For example, we helped to pack the rescue backpack for combatants. (p19) | Unequivocal |
| Knowledge and practical development | It formed some needs in wartime that they forced nurses to promote themselves. This is why nurses acquired a higher skill and knowledge … In fact they promoted. (p19) | Unequivocal |
| Increasing male entrance to nursing | Due to religious beliefs, we didn’t want women to provide care in dangerous situations and in the frontlines. For these reasons, we were encouraged to enter the nursing profession. (p19) | Credible |
| Development of educational structure for fast training of staff | In 1981, we organized an educational structure for specialized training of nurses. There were courses with different durations from 2 weeks to 2 years. With getting closer to the time of the military attack, our course duration even reduced to 2 weeks. (p20) | Unequivocal |
| Entry into the profession on a voluntary basis | Nurses at wartime did well. The reason for this success was that most of them were volunteers. (p20) | Unequivocal |
| Shaping value-based nursing | God-centered culture penetrated into our hearts. We all respected each other’s opinions … .Injuries occupied all of our minds. We did everything for ameliorating the casualties’ conditions. Thismeans sacrifice. (p20) | Credible |
| Forming a common goal in nursing | Over there, all of us looked for the efficacy of our tasks … .We all had a common goal. A condition that was created by the war made all of nurse think of an identical goal … .rescuing the injured. (p20) | Unequivocal |
| Team working | We didn’t pay attention to the label of people in the medical team … Our relationships were entirely based on mutual cooperation; and my colleagues and I were as two partners. (p21) | Unequivocal |
| Domination of enthused and fresh spirit on nursing atmosphere | It was perhaps 48 hours that I was working, but I didn’t feel fatigue. My shift was over, while I was juicy and energetic. (p21) | Credible |
| Manpower shortage crisis in the early years of the war | Lack of manpower forced all people to be trained in paramedical courses recruited to the nursing field. It was the early months of the war. The number of nurses was very low. Also, many of them were nursing students. (p21) | Unequivocal |
| New face to chemical injuries | A special feature of our warfare was the chemical warfare. When chemical injured combatants arrived, we all were scared. All were concerned; they thought it was contagious. We didn’t have special knowledge to take care of them. Over time, we could know how to care and accept them. (p21) | Unequivocal |
| Confronted with dangerous situations | Sometimes we also faced snakebite or scorpion stings. These cases annoyed the nurses. For example, many of us were infected with Leishmaniasis (Mohrram Military Operations) that it was very bad. (p21) | Unequivocal |
| Lack of specialised knowledge and skills for caring in war zones | There wasn’t specific information and education. We didn’t have special knowledge for taking care of patients. Time passed, and we learned. (p21) | Unequivocal |

Elliott B. (2015). Military nurses' experiences returning from war. Journal of advanced nursing, 71(5), 1066–1075. https://doi.org/10.1111/jan.12588

| **Finding** | **Illustrations from study** | **Evidence** |
| --- | --- | --- |
| Figuring out where I ‘fit’ in all the chaos | I would literally be standing on my front porch within 2 days of having seen some of the most horrifying trauma any human being could never imagine. And you’re trying to step into the role of being a father, husband, neighbor, friend and you can’t. Your brain cannot handle that amount of change that quickly, especially after it has been under prolonged stress...That’s the hardest part, is one minute you’re overseas, the next minute you’re standing there with your kids. (p1069) | Unequivocal |
| Feeling like it’s all so trivial now | I hated going to the stores because people would complain and I realize that my whole perspective on life just kind of changed, or beliefs that I had before kind of enhance. You get a global vision of things. You see what war’s like and you see what it does and then you stand in line and hear somebody complaining about the wait. I would set stuff down and walk out, because I was so afraid I was going to turn around and say something really not too nice. (p1070) | Credible |
| Learning to manage changes in the environment | There was a concert on TV and I had stretched out on her [sister’s] sofa and fallen asleep and they started the concert with a siren of all things and I jumped up looking around for my gas mask and my sister had to remind me that I’m back home, it’s okay. (p1070) | Credible |
| Facing the reality of multiple losses | I think you have to get to know yourself all over again. I tried to reach out to people. I lost friends....Inside I just felt completely numb and empty, like I was looking out of a mask...I felt like just getting a bulldozer and getting rid of every last friend I have and starting over with people who didn’t know me before, because then I wouldn’t go through this. (p1070) | Unequivocal |
| Working through the guilt to move forward | I think I still felt guilty and also felt a little helpless and out of control being deployed when you’re not directly there and you can’t respond to situations.... When I came home I just craved to gain control over those roles again because I felt the loss of control. (p1071) | Unequivocal |
| Serving a greater purpose in life | There’s so much terrible stuff that happened, but the amount of people that we saved...just knowing that I was a part of that...you can’t explain that to someone and explain how great you feel doing that and how the ultimate experience that the soldier that comes in is dead basically on your table and you bring them back to life, you know? (p1071) | Credible |
| Looking at life through a new lens | It means the sacrifices that you see through all means. The sacrifices of the soldiers. The sacrifices of the caregivers who are doing that role day in and day out. Sometimes in the ER just literally ankle deep in body fluids and carnage and despair. Then you take care of the locals and you just can’t believe how horrible their lives are. And it’s just an eye-opening experience. For me it was a quite a learning experience. I think you grow through that. (p1071) | Unequivocal |

Ekfeldt, B., Österberg, R., & Nyström, M. (2015). ORIGINAL PAPER. Preparing for Care in a Combat Environment. International Journal of Caring Sciences, 8(1), 1–8.

| **Finding** | **Illustrations from study** | **Evidence** |
| --- | --- | --- |
| Preparing for transition from civilian care | In a combat environment we often work in a very primitive and basic way. It is important not to complicate things, and accept the situation. (p4) | Credible |
| Preparing for work in a complex context | I thought about it in advance. How do I tell my colleagues how they should assist and what they should do? What should I report to the foreign helicopter that’s coming? How do I say this in English? I have to drill things like that. (p4) | Credible |
| Preparing to deal with anxiety | Sometimes it feels hard to be so far away. It can take eight hours by car for the nearest medically trained person to get there. So it is clear that everything is up to me if something happens. This can make you nervous sometimes. (p4) | Credible |
| Preparing by investigating one’s motives | The thing that could make me hesitate is that my son is 2.5 years old. You think about all the risks. But as my husband is an officer and has been away, he encouraged me to go. (p5) | Credible |
| Preparing by investigating one’s professionalism | The further you get in the preparatory training, the clearer the picture becomes of what it is like down there. The various elements become less strange and you feel that you have mastered them. (p5) | Credible |
| Preparing through insight into the unique meaning of the caring relationship | If anything happens to me, I hope my friends will be able to take care of me in the best way. We practiced nursing at home in training and with my buddies down in Afghanistan so I feel safe. (p5) | Credible |

Doherty, M. E., & Scannell-Desch, E. (2015). After the parade: military nurses' reintegration experiences from the Iraq and Afghanistan wars. Journal of psychosocial nursing and mental health services, 53(5), 28–35. https://doi.org/10.3928/02793695-20150406-01

| **Finding** | **Illustrations from study** | **Evidence** |
| --- | --- | --- |
| Homecoming: A Mixed Reception | My sister and her family drove from New York to meet me during my layover. It was great to see them and we had a nice visit. There was also a large group of USO [United Service Organizations] volunteers and retired military there to greet us as we came down the ramp from the jetway. It made us feel very special and appreciated. It meant the world to us to be received this way. (p31) | Credible |
| Renegotiating roles: A Family Affair | When I came back from my first deployment, I wasn’t in the right mindset to step right back into the role of working mother, housekeeper, grocery shopper, and cook. My mind just wasn’t there yet. I think my brain was a little oversaturated with the war and trauma patients I had cared for. My husband thought I would want to jump right back in, but I wasn’t up to it. I think he wanted a break from the childcare stuff. (p31) | Unequivocal |
| Painful memories of trauma | I think I had not really prepared myself for the type of trauma that I saw in Iraq. Even though I’m a trauma nurse, I had not prepared myself for all the burns, traumatic amputations, and the youth of the patients. So, when I first came back, I had a very hard time. There was a lot of burnout for me. I was just very angry about a lot of things. (p31) | Unequivocal |
| Sorting It Out:getting help | When I came home, I had trouble sleeping. I was quick to anger. I knew I needed to talk with someone, but put it off because I was afraid it would affect my career. I just didn’t want to put everything I had worked so hard for in jeopardy. (p32) | Unequivocal |
| Needing a clinical change of scenery | What frustrated me when I returned was the type of patients we take care of back in the [United States]. They are mostly older people with chronic problems. I really miss the deployment mission and trauma care we provided. It really became what I want to continue to do. I would go back in a heartbeat! Back in the [United States], nurses are tasked with doing everything. We are providing care for patients. We are sounding boards and therapists for families. We are pulled in so many directions. On deployment, you didn’t have other responsibilities clouding the mission. (p32) | Credible |
| Petty complaints and trivial whining | The kind of nursing that you go out and do on a deployment is extreme nursing, like extreme sports. When you come home, it’s kind of hard to keep up the momentum because trivial things simply do not hold the same importance. (p32) | Credible |
| Military unit or civilian job: support versus lack of support | My colleagues were supportive. They wanted to know what I did, how it went, and what I saw. It was helpful to speak of it. I think the more people hear about our experiences, the more they can understand why some of us come back with PTSD [posttraumatic stress disorder] or “compassion fatigue. (p32) | Unequivocal |
| Family and social networks: support versus lack of support | I mailed informational packets with a letter to the principal of the high school to give to my son’s teachers in September. The letter explained I was going to deploy and that I would not be back until January. Well, none of my son’s teachers received the packets. In fact, half of his teachers didn’t even know that I was deployed. My son became very depressed.... After therapy, my son is doing a lot better now, but I’m very upset that his school dropped the ball and didn’t get the information to [his] teachers. (p33) | Unequivocal |
| Reintegration: a new normal | When I come home from deployments, the key is not trying to do too much too soon. I learned that from multiple deployments. I learned that it was not always good to go right home to family. If you can take a week to decompress and be by yourself, do it! I must say I have never been a person with a temper, but it has probably gotten a little shorter after having three deployments. (p33) | Credible |

Rivers, F. M., Gordon, S., Speraw, S., & Reese, S. (2013). U.S. Army nurses' reintegration and homecoming experiences after Iraq and Afghanistan. Military medicine, 178(2), 166–173. https://doi.org/10.7205/milmed-d-12-00279

| **Finding** | **Illustrations from study** | **Evidence** |
| --- | --- | --- |
| Command support: no one Cared | [Nurses] would sit around and talk and they would complain that no one cared that they had deployed; no one acknowledged them. (p170) | Unequivocal |
| Check the blocks | Reintegration is very important. It is not just a matter of checking the block; it is a matter of making sure it is done correctly ... people are not going to be up front in the beginning [if they are having problems]. They are not going to say ‘I am a basket case.’ If you are going to only screen people at 90 days, 60 days, or 120 days ... you might as well not even check those blocks ... Have some [other] type of follow-up ... some kind of group that would say ... ‘I am following up with you’ ... [just] getting a phone call from somebody. (p170) | Unequivocal |
| The stress of coming home | The stress of being back in America is huge! Having to multitask again ... . It was hard to come back initially, transitioning ... I can’t explain why ... having to choose and make decisions ... going to the grocery store, big bright lights, aisles of food to choose from (not just one thing to choose from and pick but a variety), the cereal aisle for example ... what cereal am I going to buy. (p170) | Unequivocal |
| They don’t understand | You can’t talk to your family about it ... unless they have deployed or served in the military and went through [it] ... maybe Vietnam or Desert Storm, they really don’t understand. (p171) | Unequivocal |
| It changes you | It just changes you ... it probably took about 2 years before I could really say I’m...okay, I’m different, I’ll never be the same because of the experience. (p171) | Unequivocal |

Goodman, P., Edge, B., Agazio, J., & Prue-Owens, K. (2013). Military nursing care of Iraqi patients. Military medicine, 178(9), 1010–1015. https://doi.org/10.7205/MILMED-D-13-00055

| **Finding** | **Illustrations from study** | **Evidence** |
| --- | --- | --- |
| Expanding practice | I enjoy the opportunity to share my knowledge and experience and have actually conducted in services for nurses. For me, that is rewarding—to have the chance to be a teacher and trainer. (p1012) | Unequivocal |
| Ethical dilemmas | How can we possibly leave these patients in their care. Are they going to be doing the same things that we are doing? I kind of feel guilty. (p1012) | Credible |
| Cultural divide | I have a female [patient] with chest pain. I couldn’t do an EKG because she did not want me to see her chest because of her religion or whatever. I am here to respect their culture.... but the female-female, male-male thing. I am not used to that. It is a learning experience, to  respect cultural ways. (p1013) | Credible |

Scannell-Desch, E., & Doherty, M. E. (2010). Experiences of U.S. military nurses in the Iraq and Afghanistan wars, 2003-2009. Journal of nursing scholarship : an official publication of Sigma Theta Tau International Honor Society of Nursing, 42(1), 3–12. https://doi.org/10.1111/j.1547-5069.2009.01329.x

| **Finding** | **Illustrations from study** | **Evidence** |
| --- | --- | --- |
| Deploying to war | I volunteered for Afghanistan. I had been in the Navy for 26 years. Many people had said to me, “I bet you’ve been on this ship, and that ship, since you’ve been in the Navy for a very long time.” Well, I had never been on a ship or seen a war, so it was a little embarrassing to be in the Navy for that length of time and never experienced a war, or never served on a ship. So the Navy found me a position in Afghanistan in a place called Mazar-el-Sharif as an individual augmentee on a 12-person mentoring team. (p7) | Credible |
| Remembrance of war: most chaotic scene | We had three young soldiers brought into our emergency room. A rocket-propelled grenade had ripped through their Humvee a little below sitting height, and it traumatically amputated every one of their six legs. One soldier was unconscious, another was stable, and the third was like a squirrel set out in the middle of the road and didn’t know which way to go. He was panic stricken, sat up on the gurney and look down where his legs used to be and screamed bloody murder. “Oh, my God, I don’t have any legs.” He threw himself back on the gurney and just continued screaming at the top of his lungs. I had to leave the tent and go outside and throw-up. It made me physically sick to see this poor child the way he was. It took hours to calm him down so we could fly him to Germany. (p8) | Unequivocal |
| Nurses in harm’s way: more than I bargained for | I was hit by a mortar blast. I was hit with shrapnel in the neck and also have right-sided hearing loss. I had just left the trauma unit and was walking through the galley when the compound was hit. I was medevaced back in one of the Air Force’s flying hospitals. (p9) | Credible |
| Kinship and bonding | There was friendship. I still have some really good friends I met over there. I met another nurse before we deployed, who ended up being my roommate and best friend. You end up depending and relying on each other so much over there. (p9) |  |
| My war stress: I’m a different person now | I recently started going to a V.A. hospital because I’m having anxiety and panic issues at work. I found my reserve for empathy and compassion was drained. Anything could set me off, make me mad and verbal. Before I went to Iraq, I was a laid-back person. I didn’t let a lot bother me. Now, I’m increasingly bothered and it seems to only happen at work. (p10) | Credible |
| Professional growth: expanding my skills | The Army was building a new hospital in Anbar province, and this was during the surge, so they needed me there. I was a nurse practitioner, but they needed me in surgery as first assistant. I said, “I don’t do ORs, I don’t even know what an OR looks like!” They said, “Don’t worry, we’ll teach you!” So, they taught me. With all the amputations coming in, I was very busy. (p10) | Credible |
| Listen to me: advice to deploying nurses | “I’ve talked to nurses, and I encourage them to volunteer for Iraq. I tell them, ‘Honey, if you want an experience that you will never have as long as you live, go to Iraq. (p11) | Credible |

Agazio J. (2010). Army nursing practice challenges in humanitarian and wartime missions. International journal of nursing practice, 16(2), 166-175. https://doi.org/10.1111/j.1440-172X.2010.01826.x

| **Finding** | **Illustrations from study** | **Evidence** |
| --- | --- | --- |
| Required nursing competencies in wartime and military operations other than war | The biggest skills you need to know is that you have a very limited amount of resources. You can’t just keep opening up you know, a triple lumen pack if it gets contaminated. You just don’t have a lot of IV fluids. You have to be very careful on how you waste supplies, which I think we’re not used to in the military at a medical centre. We’re always used to running and getting another pack of whatever we need if something happens. But in the deployed austere environment you will find that your resupply is sporadic and your blood supply is very low, so you have to be careful. (p169) | Credible |
| Deployed environment | When we first got there, we drew a set [of supplies] that had been in storage in a warehouse somewhere in the desert. It was all brand new equipment DEPMEDS-wise [Deployable Medical System], which was nice, dealing with that. But we had supply issues because everything was fried. Rubber and plastic pieces were crispy and brittle. Gloves turned to powder in your hand when you tried to put them on. So we had supply issues initially and getting the supply lines going was a long slow process. (p169) | Credible |
| Patient care demands | Most of the trauma we took care of was minor trauma. Lacerations and that kind of stuff and a lot of sport injuries, ankles and things like that were probably the biggest thing that we saw injury wise. (p169) | Unequivocal |
| Getting down to basics | The field mechanical ventilation machine was definitely different. The field EKG machine, just your cardiac monitoring, your A-lines. You had to rely on some of your innovated ways, like how do you ensure that your transducer is level before you take any reading for your CVPs [central venous pressure] and stuff? Well you don’t have a level available to you, so you take a blood tube, fill it with some water so there is an air bubble and you tie it to a string, and you just make your own little level device. So you have to be innovative in the way you think about doing business. (p170) | Unequivocal |
| Level of care | Horrific injuries where you got flesh hanging all over the place, big bullet holes. I’ve never seen anybody shot before and just seeing a bullet go in one side where you get this quarter size injury hole and then you cut and see the back of somebody’s calf, for instance, and there’s this four or five inch cross exit wound. It’s just amazing the damage that these things can do. We had one mass casualty with two patients: one who got shot in the arm, right in his triceps and the bullet wound up travelling all the way up and lodging in his head. This guy died. The other guy in the same event who got shot in the chest but it hit him in the back of his chest under his scapula and then didn’t even enter the rib cages, travelled right along the outside of the ribs and came out underneath his left arm and he was fine. You would think that the guy who got shot in the arm would be the guy who is okay but he died and the other guy got shot in the chest and wound up surviving. It’s just different; you have to be prepared for all different types of injury. (p171) | Unequivocal |
| Training | We should have been prepared, better prepared to do that. They don’t spend a lot of time teaching nurses and physicians that. It’s just not done. (p172) | Credible |

Rushton, P., Scott, J. E., & Callister, L. C. (2008). "It's what we're here for:" nurses caring for military personnel during the Persian Gulf Wars. Nursing outlook, 56(4), 179–186.e1. https://doi.org/10.1016/j.outlook.2008.03.010

| **Finding** | **Illustrations from study** | **Evidence** |
| --- | --- | --- |
| Giving humanitarian service | There are a lot of things we do to try to win the war on terrorism, like medical humanitarian missions. The civil affairs team [rebuilt] schools, wells, and roads. There was a big push to do these things to win the hearts and minds of countries at risk for having an insertion of Al Qaeda. You were contributing to that mission by participating in humanitarian work. (p180) | Unequivocal |
| Dealing with supply issues | We needed an overhead trapeze. I had my nursing fundamentals book with a picture of a trapeze. I showed it to a Seabee. He made a special bed with a trapeze on it for an amputee. (p181) | Credible |
| Acting as a prepared cohesive unit | We were very busy with broken bones, twisted ankles, lacerations, deaths from suicide, which weren’t pretty. We had Humvee rollovers [with no] seatbelts. People did wear their flaks and Kevlars occasionally. We had construction site injuries; deep lacerations, skin evulsions and getting rings caught, degloving injuries. That was before the war actually started. (p181) | Credible |
| Dealing with issues related to patient disposition | All of the nurses had such ownership that none of us would leave if there was a casualty. All of us slept in the sick call tent so that we would be right next to the admitting area if a Humvee with casualties drove up. We were very selfless when it came to that because it’s “what we’re here for” and forget about trying to sleep or eat. We’re here for the guys. (p181) | Credible |
| Caring for non-combatants | Our work began when 3 recruits were transferred to ICU after pneumonia [escalated to] empyema and pleural effusion. The laboratory and radiology departments received 2 weeks’ work in 4 days. (p181) | Unequivocal |
| Sacrificing physical safety | We had some incoming scud missiles. We went through the scud alerts. I worked night shift during the first 2 days of the war and 2 hours of continuous sleep was a lot. I’m not complaining. We certainly had it better than the folks crossing the border and heading north. I got to the point where I just slept in my chemical-biological gear without the mask. Every 2 hours we would hit the bunkers and sit there, then go back and go to sleep. On one of our last scud alerts, our patriot battery engaged a missile just a mile away. I didn’t realize how close it was. We didn’t know it was on base. I watched the local news and they said, “Army headquarters in Kuwait almost gets attacked.” I’m looking at the news and they’re showing the command room and I’m going, “That’s me.” (p182) | Unequivocal |
| Experiencing personal and professional change | When I got back I did not want to share anything. If somebody wasn’t there, they couldn’t understand it. The intelligence briefing beforehand was about how you might get shot and how you have to strap on all your chemical gear when you land in the desert. That was enough to scare the crap out of us. You don’t talk a lot about that stuff. We went through about a month where everybody in our squadron didn’t really say anything until one person said, “Are you having any difficulties now that we’re back?” It turns out that everybody is having some difficulty. (p182) | Credible |
| Caring for other members of the health care team. | Whenever we drove in convoy, people would reach into your vehicle and try to steal things. As they’re reaching in you’d swat them, and that was your protection basically. We all had to carry guns. You always had your weapon strapped to your chest. I and the other flight nurse ended up back to back in the vehicle facing our respective window because these people were just converging on our vehicle and they were grabbing at anything that wasn’t tied down. They popped the back of the vehicle and started scrambling in. This whole time, I kept thinking, “Please don’t let me have to pull this gun off my chest and start using it.” The next thing I hear is the nurse who’s got her back to me saying, “That guy over there has a knife.” I looked around and this guy has lifted up his shirt to show that he’s got this huge machete stuck down his pants. I‘’m like, “Oh my goodness, please, please just let us get through here.” The traffic finally cleared. What seemed like an eternity was probably only about 10 minutes. We got back to the base and I remember that incredible adrenaline surge of thinking, “We made it. I’m glad we’re here [at the base]. I will never do it again.” But, I am so glad I had that experience because it was so incredible. (p183) | Credible |
| Caring about one’s country | The United States has always been the country that has helped. Maybe this will turn out to be a bad thing, but we’ve always valued life, and that’s why we’re involved there. (p183) | Credible |
| Feeling cared for by countrymen. | We would get messages from home saying, ‘We’re so supportive . . . We love what you guys are doing for us . . . We’re thinking about you guys.” It added a lot of validation to what we were doing. When you hear people being so supportive and not believing in the negative media, then it makes what you’re doing out there meaningful. The military contracted out some civilian flights to make the trip. The staff on the flight, the pilots and the flight attendants, were volunteers, people who wanted to be involved with transporting the troops. It was pretty amazing. (p183) | Credible |
| Caring for patients. | You quit being scared after a while. I got letters from elementary school children that asked about fear. When I wrote them back I said that the thing I was most afraid of is that there would be somebody who comes in who we couldn’t help. That was the truth. I wasn’t scared for myself anymore. (p184) | Credible |

Griffiths, L., & Jasper, M. (2008). Warrior nurse: duality and complementarity of role in the operational environment. Journal of advanced nursing, 61(1), 92–99. https://doi.org/10.1111/j.1365-2648.2007.04469.x

| **Finding** | **Illustrations from study** | **Evidence** |
| --- | --- | --- |
| A Changing World | You were almost detached from the whole situation, because we were 100ks back off the frontline, so apart from the alarms going off for the scud attacks that was about the only thing that really made you think ‘Oh, maybe I could be in danger’ . (p95) | Credible |
| The Human Impact of War | The worse thing was seeing all the ruined houses and kids on the side of the street begging for food. Seeing the way the Serb militia had daubed the walls with some of the atrocities they had committed in the houses. (p95) | Credible |
| The Interface of Professions | It’s job role at the end of the day. I’m not just answerable to the military, I’m answerable to the civilian side also and at the end of the day the c**p stops at my door! (p95) | Credible |
| Value Laden Image | …the padre is fantastic at welfare. However, you see him as the padre, you don’t see him as a Captain, Major or whatever. That is probably why we still have this issue of credibility, where they don’t see us as Army people – they see us as people who are going to patch them up when they are injured. (p96) | Credible |
| Warrior Nurse | We set up, with the Gurkhas, a disco for the under 10s in a local village. We were on the way back, we had broken curfew, but had permission to be out late. We were in an open top vehicle and we only had 10 live rounds amongst us. The armoured vehicle with the Gurkhas that was supposed to be protecting us seemed like it was at least a mile behind. Suddenly we came to this civilian roadblock. I leaned over, out of the vehicle, to see what was going on and it was an armed roadblock. At the time I just thought, ‘Oh my God! Me or them!’ I just went, ‘Well make yourself ready’. I basically flipped my safety (catch) off and switched to automatic (fire). I thought, ‘I’ve got 10 rounds, I will spray’. As we were getting ready, the armoured vehicle came and ploughed them (the civilian roadblock) off the road…I never thought I would just flip my safety (catch) off and point my weapon and actually use it. I still don’t know if I would, to this day, but I was prepared to at that point. (p96) | Unequivocal |
| Their Story | I’m comfortable with who I am and what uniform I wear...it allows you to concentrate on what you are actually there to do. What we are interested in is ‘Can you do your professional job in the environment of the field?’ I think that is a key skill for the military nurse. (p97) | Credible |

Scannell-Desch E. A. (2005). Lessons learned and advice from Vietnam war nurses: a qualitative study. Journal of advanced nursing,49(6), 600–607. https://doi.org/10.1111/j.1365-2648.2004.03335.x

| **Finding** | **Illustrations from study** | **Evidence** |
| --- | --- | --- |
| Advice about journaling | My journal helped me remember people, what I was thinking in those days, and how I reacted to situations. I look back on it now, and it was very useful in helping me put Vietnam in perspective. (p603) | Unequivocal |
| Advice about training | Ever since that time, if I have anything to do with training, I try to make it as realistic as possible. Many times I’ll tell our folks, the only reason we exist in the peacetime arena is to learn, so we can transfer that learning to the wartime environment. (p603) | Credible |
| Advice about caring for yourself | I think I would advise them first of all to know themselves. To know what works best for them as far as coping mechanisms because it is a rough, stressful area’, exclaimed an Air Force nurse. (p604) | Credible |
| Advice about support systems | I think that support systems are very important. To have people that are supportive of you, and that you know you are not alone, that other people are out there feeling the same things. (p604) | Unequivocal |
| Advice about talking about your experiences | We just closed it off. I couldn’t talk to people at the time. I didn’t want to show my own inadequacies to other people. (p604) | Unequivocal |
| Advice about understanding the mission | They need to understand that the purpose of the military is to support, protect, and defend a country’s national security interests. This can involve war, and military nurses have to be ready for war. In wars, terrible things happen to young soldiers, it is not nice, but it is the reality they will have to deal with. It takes a special kind of nurse to be a military nurse. (p605) | Credible |
| Advice about lack of preparation | I was not prepared emotionally, physically, or professionally to deal with the kinds of patients we had to deal with in Vietnam. (p605) | Unequivocal |

Cox CW. (2005). Shipboard nursing on aircraft carriers: the perceptions of twelve Navy nurses. Nursing Outlook, 53(5), 247–252.

| **Finding** | **Illustrations from study** | **Evidence** |
| --- | --- | --- |
| Experiencing the best but toughest job the Navy has to offer its nurses | It was probably the most challenging, demanding, rewarding, and exciting job I’ll ever have in the Navy . . . . You really got a sense of what the Navy was all about . . . when you’re out to sea. You can see the Navy working, launching jets and doing underway replenishments and seeing every aspect of the Navy at once from every sailor doing their job . . . so it was really kind of neat to be a part of something bigger and you felt a sense of mission and you felt your contribution to that mission. (p249) | Credible |
| Ensuring readiness | At one point, I kind of reached a breaking point and tried to be departmental training coordinator, ward nurse, ICU nurse, training team leader, credentials nurse, QA nurse, and try[ing] to do health promotions, and wear all the hats, and try to have balance. It was really difficult. (p249) | Credible |
| Being one-of-one | I had no corpsmen that had any inpatient experience whatsoever. You are the only ICU nurse, which means when I [had] my two ICU beds full, I was literally catching a catnap on the floor between the two patients. (p250) | Credible |
| Operating constantly in an environment of uncertainty | I think the biggest challenge of a ship’s nurse is developing your corpsmen . . . . No matter who you are, no matter how good you are, 24 hours a day, seven days a week, at some point, you’re going to have to get sleep and some point, you’re going to have to shower, [and] you’re going to have to eat. So . . . at some point, you’re going to have to leave the ward, and trust the ward, and the patients, to the corpsmen. If you can’t do that . . . you’re not going to make it because physically, Mother Nature says you have to eat, you have to sleep, you have to have socialization, or you just won’t do well. (p250) | Unequivocal |
| Having 2 families | I didn’t have a lot of ship savvy when I got there. But these folks saw . . . somebody who cared about the ship . . . . So here’s somebody who they didn’t just see as a nurse .... There’s that other aspect of it: You’re a [nurse] . . . but you’re also. . . a Naval citizen. You’re going there to be part of that ship. And you’re a shipmate. So they never just blew me off because I was a nurse. (p250) | Credible |
| Making the job better for the next generation | I was literally running almost from one drill set, zipping through the medical department, getting into the ICU. I had a box of 10 syrettes of demerol in 1 pocket and a box of 10 syrettes of morphinein the other pocket as I’m running around the ship. And I’d come down into [the] Medical [Department]. I had the different names . . . labeled on the tubex and then I would give them a little bit of IV [intravenous] push med [medication] for pain. And then, boom! I was out the door again after documenting . . . that I gave something. And then, boom! I was out running more drills or attending more meetings. That was absolutely ludicrous. I couldn’t have been getting but maybe 3 hours of sleep every night. That’s another reason why you kind of need another nurse. (p251) | Credible |

Scannell-Desch E. A. (1996). The lived experience of women military nurses in Vietnam during the Vietnam War. Image--the journal of nursing scholarship, 28(2), 119–124. https://doi.org/10.1111/j.1547-5069.1996.tb01203.x

| **Finding** | **Illustrations from study** | **Evidence** |
| --- | --- | --- |
| Facing moral and ethical dilemmas | Sometimes I look back and wonder if these guys have cursed us for saving them because they have handicaps, like the spinal cord injuries or the one that lost several limbs. It bothered me that some of the policies and politics would not allow the fighting to be conducted in a way it was supposed to be. (p123) | Credible |
| Giving of oneself | It was a difficult time because of the types of patients we cared for ... the distance from home, but there was more to it than that. You were a mother, you were a girlfriend, you were a sister, you were a nurse. We had the feeling that every GI who came through that door-they were our neighbors and brothers, and we had to save them. (p123) | Credible |
| Improvising | I could make pizza in a frying pan, in fact, I could cook a full-course dinner in a frying pan. (p123) | Unequivocal |
| Feeling out of place | They didn’t sell much in the way of female supplies in the PX at Long Binh post. We mostly had to rely on family to send us tampons, hair spray, and stuff like that. (p123) | Credible |
| Lacking privacy | I remember one night, a nurse came down and asked if she could use my room. I asked her what she had in mind, and she said she was looking for a place to cry. There just wasn’t any place to be alone. (p123) | Credible |
| Re-creating home | We had Thanksgiving dinner at our place. We had everything we would ordinarily have. We sent someone to Saigon to get everythmg and it worked. We did have a turkey, mashed potatoes, and cranberry sauce and some vegetables. (p123) | Credible |
| Bonding | I think that I probably have never felt the same way about a group of people as I did about that group; they were the best! There was a bond I had never felt before. We each had a role to play and each one was as distinctly important as the next. (p123) | Unequivocal |
